# Modelling health outcomes of a decade of HIV, malaria and tuberculosis initiatives, Malawi

**DOI:** 10.2471/BLT.24.292439

**Published:** 2025-03-07

**Authors:** Tara Danielle Mangal, Margherita Molaro, Dominic Nkhoma, Tim Colbourn, Joseph H Collins, Eva Janoušková, Matthew M Graham, Ines Li Lin, Emmanuel Mnjowe, Tisungane E Mwenyenkulu, Sakshi Mohan, Bingling She, Asif U Tamuri, Pakwanja D Twea, Peter Winskill, Andrew Phillips, Joseph Mfutso-Bengo, Timothy B Hallett

**Affiliations:** a MRC Centre for Global Infectious Disease Analysis, Jameel Institute, School of Public Health, Imperial College London, Exhibition Rd, South Kensington, London SW7 2AZ, England.; b Health Economics and Policy Unit, Kamuzu University of Health Sciences, Lilongwe, Malawi.; c Institute for Global Health, University College London, London, England.; d UCL Centre for Advanced Research Computing, University College London, London, England.; e National Tuberculosis and Leprosy Elimination Program, Ministry of Health, Lilongwe, Malawi.; f Centre for Health Economics, University of York, York, England.; g Department of Planning and Policy Development, Ministry of Health and Population, Lilongwe, Malawi.

## Abstract

**Objective:**

To estimate the outcome of programmes on human immunodeficiency virus and acquired immunodeficiency syndrome (HIV/AIDS), tuberculosis and malaria in Malawi across multiple health domains.

**Methods:**

We used an integrated epidemiological and health system model to estimate the impact of HIV/AIDS, tuberculosis and malaria programmes in Malawi from 2010 to 2019. We incorporated interacting disease dynamics, intervention effects and health system use in the model. We examined four scenarios, comparing actual programme delivery with hypothetical scenarios excluding the health programmes individually and collectively.

**Findings:**

From 2010 to 2019, an estimated 1.08 million deaths and 74.89 million disability-adjusted life years were prevented by the HIV/AIDS, tuberculosis and malaria programmes. An additional 15 600 deaths from other causes were also prevented. Life expectancy increased by 13.0 years for males and 16.9 years for females. The programmes accounted for 18.5% (95% uncertainty interval, UI: 18.2 to 18.6) of all health system interactions, including 157.0 million screening and diagnostic tests and 23.2 million treatment appointments. Only 41.5 million additional health worker hours (17.1%; 95% UI: 15.9 to 17.4%) of total health worker time) were needed to achieve these gains. The HIV/AIDS, tuberculosis and malaria programmes required an additional 120.7 million outpatient appointments, which were offset by a net decrease in inpatient care (9.4 million bed-days) that would have been necessary in their absence.

**Conclusion:**

HIV/AIDS, tuberculosis and malaria programmes have greatly increased life expectancy and provided direct and spill-over effects on health in Malawi. These investments reduced the burden on inpatient and emergency care, which requires more intensive health worker involvement.

## Résumé

**Objectif:**

Évaluer les résultats de programmes de lutte contre le virus de l’immunodéficience humaine et le syndrome d’immunodéficience acquise (VIH/SIDA), la tuberculose et le paludisme au Malawi dans plusieurs domaines de la santé.

**Méthodes:**

Nous avons utilisé un modèle épidémiologique intégré et un modèle de système de santé pour évaluer l’impact des programmes de lutte contre le VIH/SIDA, la tuberculose et le paludisme au Malawi entre 2010 et 2019. Nous avons intégré au modèle la dynamique des maladies en interaction, les effets des interventions et le recours au système de santé. Nous avons examiné quatre scénarios, comparant la mise en œuvre réelle des programmes à des scénarios hypothétiques excluant les programmes de santé individuellement et collectivement.

**Résultats:**

De 2010 à 2019, les programmes de lutte contre le VIH/SIDA, la tuberculose et le paludisme ont permis d’éviter environ 1,08 million de décès et 74,89 millions d’années de vie corrigées de l’incapacité. En outre, 15 600 décès dus à d’autres causes ont été évités. L’espérance de vie a augmenté de 13,0 ans pour les hommes et de 16,9 ans pour les femmes. Ces programmes ont représenté 18,5% (intervalle d’incertitude à 95%: 18,2–18,6%) de toutes les interactions avec le système de santé, dont 157,0 millions de tests de dépistage et de diagnostic et 23,2 millions de rendez-vous pour traitement. Seules 41,5 millions d’heures de travail supplémentaires pour les professionnels de la santé (17,1% ; intervalle d’incertitude à 95%: 15,9–17,4%) du temps total consacré par les professionnels de la santé) ont été nécessaires pour parvenir à ces progrès. Les programmes de lutte contre le VIH/SIDA, la tuberculose et le paludisme ont nécessité 120,7 millions de consultations externes supplémentaires, qui ont été compensées par une diminution nette des soins hospitaliers (9,4 millions de journées d’hospitalisation) qui auraient été nécessaires en l’absence de ces programmes.

**Conclusion:**

Les programmes de lutte contre le VIH/SIDA, la tuberculose et le paludisme ont considérablement augmenté l’espérance de vie et ont eu des effets directs et indirects sur la santé au Malawi. Ces investissements ont permis de réduire la charge des soins hospitaliers et des soins d’urgence, qui nécessitent une intervention plus intensive du personnel de soins de santé.

## Resumen

**Objetivo:**

Estimar el impacto de los programas sobre el virus de la inmunodeficiencia humana y el síndrome de inmunodeficiencia adquirida (VIH/sida), la tuberculosis y la malaria en Malaui en diversos ámbitos de la salud.

**Métodos:**

Se utilizó un modelo integrado de epidemiología y sistema de salud para estimar el impacto de los programas de VIH/sida, tuberculosis y malaria en Malaui entre 2010 y 2019. El modelo incorporó las dinámicas de interacción entre enfermedades, los efectos de las intervenciones y el uso del sistema de salud. Se examinaron cuatro escenarios, comparando la implementación real de los programas con escenarios hipotéticos en los que se excluían individual y colectivamente estos programas de salud.

**Resultados:**

Entre 2010 y 2019, los programas de VIH/sida, tuberculosis y malaria evitaron aproximadamente 1,08 millones de muertes y 74,89 millones de años de vida ajustados por discapacidad. Asimismo, se previnieron 15 600 muertes adicionales por otras causas. La esperanza de vida aumentó en 13,0 años para los hombres y 16,9 años para las mujeres. Los programas representaron el 18,5% (intervalo de incertidumbre [II] del 95%: 18,2 a 18,6) de todas las interacciones con el sistema de salud, incluidas 157,0 millones de pruebas de cribado y diagnóstico, así como 23,2 millones de citas para tratamiento.  Solo fueron necesarias 41,5 millones de horas adicionales de trabajo del personal sanitario (17,1%; II del 95%: 15,9 a 17,4% del tiempo total del personal) para lograr estos avances. Los programas de VIH/sida, tuberculosis y malaria requirieron 120,7 millones de consultas ambulatorias adicionales, compensadas por una reducción neta en la atención hospitalaria (9,4 millones de días de hospitalización) que habría sido necesaria en su ausencia.

**Conclusión:**

Los programas de VIH/sida, tuberculosis y malaria han aumentado significativamente la esperanza de vida y han generado efectos directos y colaterales positivos en la salud de Malaui. Estas inversiones redujeron la carga de la atención hospitalaria y de emergencia, que requiere una mayor implicación del personal sanitario.

## ملخص

الغرض تقدير نتائج البرامج الخاصة بفيروس نقص المناعة البشرية، ومتلازمة نقص المناعة البشرية المكتسبة (الإيدز)، والسل، والملاريا في ملاوي عبر مجالات صحية متعددة.

الطريقة لقد استخدمنا نموذجًا متكاملًا للنظم الوبائية والصحية لتقدير تأثير برامج فيروس نقص المناعة البشرية المكتسبة (الإيدز)، والسل، والملاريا في ملاوي من عام 2010 إلى عام 2019. قمنا بدمج ديناميكيات المرض المتفاعلة، وتأثيرات التدخل، واستخدام النظام الصحي في النموذج. لقد اختبرنا أربعة سيناريوهات، وقمنا بمقارنة بين تقديم البرنامج الفعلي والسيناريوهات الافتراضية باستثناء البرامج الصحية بشكل فردي وجماعي.

النتائج من عام 2010 إلى عام 2019، تم منع ما يقدر بنحو 1.08 مليون حالة وفاة، و74.89 مليون سنة من معدلات سنوات العمر المعدلة بالإعاقة، من خلال برامج مكافحة فيروس نقص المناعة البشرية، والإيدز، والسل، والملاريا. كما تم أيضًا منع 15600 حالة وفاة إضافية لأسباب أخرى. وزاد متوسط العمر المتوقع بمقدار 13.0 سنة للذكور، و16.9 سنة للإناث. وشكلت البرامج %18.5 (فاصل عدم ثقة %95: 18.2 إلى 18.6) من جميع تفاعلات النظام الصحي، بما في ذلك 157.0 مليون اختبار فحص وتشخيص، و23.2 مليون موعد علاج. ولم تكن هناك حاجة إلا إلى 41.5 مليون ساعة إضافية للعاملين في القطاع الصحي (%17.1؛ فاصل عدم ثقة %95: 15.9 إلى %17.4) من إجمالي وقت العاملين في القطاع الصحي) لتحقيق هذه المكاسب. وتطلبت برامج فيروس نقص المناعة البشرية/الإيدز والسل والملاريا 120.7 مليون موعد إضافي للمرضى الخارجيين، وهو ما تم تعويضه بانخفاض صافٍ في رعاية المرضى الداخليين (9.4 مليون يوم إشغال سريري)، كان ضروريًا في غياب هذه البرامج.

الاستنتاج لقد أدت برامج مكافحة فيروس نقص المناعة البشرية/الإيدز، والسل، والملاريا، إلى زيادة كبيرة في متوسط العمر المتوقع، كما أحدثت تأثيرات مباشرة وغير مباشرة على الصحة في ملاوي. وقد أدت هذه الاستثمارات إلى تخفيف العبء على رعاية المرضى الداخليين، والرعاية الطارئة، وهو ما يتطلب مشاركة أكثر فعالية من جانب العاملين في القطاع الصحي.

## 摘要

**目的:**

评估马拉维在多个卫生领域推行人体免疫缺陷病毒和获得性免疫缺陷综合征(艾滋病毒/艾滋病)、结核病和疟疾防控项目的结果。

**方法:**

我们综合使用流行病学和卫生系统模型来评估 2010 年至 2019 年期间马拉维艾滋病毒/艾滋病、结核病和疟疾防控项目的影响。我们将相互作用的疾病动态、干预效果和卫生系统介入等因素都纳入了模型之中。我们研究了四种情景,将实际的项目实施情况与那些分别和综合排除了卫生项目的假设情景进行了比较。

**结果:**

从 2010 年到 2019 年,艾滋病毒/艾滋病、结核病和疟疾防控项目预计避免了大约 108 万人死亡并减少了 7,489 万年的伤残调整生命年。同时还避免了另外 15,600 人因其他原因而致死。男性的预期寿命延长了 13.0 岁,而女性的预期寿命则延长了 16.9 岁。在所有卫生系统交互作用(其中包括 1.57 亿次筛查和诊断检测,以及 2,320 万次治疗预约)中,这些防控项目占比 18.5%(95% 不确定区间 (UI):18.2% 至 18.6%)。仅需另外花费卫生工作者 4,150 万个小时(占卫生工作者总时间的 17.1%,95% UI:15.9% 至 17.4%),即可获得上述效果。艾滋病毒/艾滋病、结核病和疟疾防控项目额外需要 1.207 亿人次的门诊预约,而这一数量被住院护理的净减少量(940 万个床日)所抵消,因为如果没有这些防控项目,住院护理需求将会更高。

**结论:**

艾滋病毒/艾滋病、结核病和疟疾方案大幅延长了预期寿命,并对马拉维的整体健康情况产生了直接和溢出效应。此类投入减轻了原本需要更多卫生工作者参与的住院治疗和急诊护理的负担。

## Резюме

**Цель:**

Оценить для ряда областей здравоохранения в Малави результаты внедрения программ по борьбе с вирусом иммунодефицита человека и синдромом приобретенного иммунодефицита (ВИЧ/СПИД), туберкулезом и малярией.

**Методы:**

Авторы использовали объединенную модель эпидемиологии и системы здравоохранения для оценки влияния программ по борьбе с ВИЧ/СПИДом, малярией и туберкулезом, которые проводились в Малави с 2010 по 2019 год. В модель были включены: динамика взаимодействующих заболеваний, эффекты вмешательств и использование системы здравоохранения. Рассматривались четыре сценария, в которых сравнивалось фактическое проведение программ с гипотетическими сценариями, из которых исключались индивидуальные и коллективные программы здравоохранения.

**Результаты:**

В период с 2010 по 2019 год за счет программ по борьбе с малярией, туберкулезом и ВИЧ/СПИДом удалось предотвратить около 1,08 миллиона смертей и потерю 74,89 миллиона лет жизни с поправкой на инвалидность. Кроме того, было предотвращено 15 600 смертей от других причин. Ожидаемая продолжительность жизни повысилась на 13,0 года для мужчин и 16,9 года для женщин. На долю программ пришлось 18,5% (95%-й доверительный интервал, ДИ: от 18,2 до 18,6) всех взаимодействий с системой здравоохранения, включая 157,0 миллиона случаев скрининга и проведения диагностических тестов, а также 23,2 миллиона назначений лечения. Для достижения этих целей понадобилось всего 41,5 миллиона дополнительных часов работы сотрудников здравоохранения (17,1% от общего количества рабочего времени в отрасли, 95%-й ДИ: от 15,9 до 17,4%). Программы по борьбе с ВИЧ/СПИДом, туберкулезом и малярией потребовали дополнительно 120,7 миллиона амбулаторных посещений, что взамен привело к сокращению госпитализаций на 9,4 миллиона койко-дней, которые были бы необходимы в противном случае.

**Вывод:**

Программы по борьбе с малярией, туберкулезом и ВИЧ/СПИДом резко повысили ожидаемую продолжительность жизни и оказали прямое и даже более высокое дополнительное влияние на здоровье населения в Малави. Эти вложения сократили бремя госпитализаций и услуг неотложной помощи, для чего требуется более интенсивное привлечение работников здравоохранения.

## Introduction

Between 2010 and 2020, Malawi’s substantial investments in human immunodeficiency virus and acquired immunodeficiency syndrome (HIV/AIDS), tuberculosis and malaria programmes have significantly reduced disease burden.^1^ This decade of targeted interventions saw remarkable progress in public health through comprehensive testing, treatment and preventive services.

Widespread access to antiretroviral therapy (ART) for HIV has improved life expectancy and health for individuals living with HIV/AIDS in Malawi. The adoption of the Joint United Nations Programme on HIV/AIDS (UNAIDS) 90–90–90 targets resulted in a decrease in new HIV infections between 2010 and 2020, from 58 000 to 17 000 cases annually, and a reduction in AIDS deaths from 37 000 to 13 000.^2^ Coverage of prevention of mother-to-child transmission services was estimated to reach 96.3% for maternal ART and 92.3% for infant prophylaxis by 2021.^3^ Tuberculosis control efforts have improved case detection rates from 42.0% (21 152/50 362) in 2010 to 56.0% (14 977/26 744) in 2020, while the tuberculosis treatment success rate for drug-sensitive strains reached 89.0% (13 330/14 977).^4^ Insecticide-treated bednets and effective antimalarial drugs have substantially reduced malaria morbidity and mortality. In 2020, malaria incidence decreased to 219 cases per 1000 people, from 381 cases per 1000 people in 2010. Similarly, the malaria mortality rate dropped to 38 deaths per 100 000 people, compared with 73 deaths per 100 000 people in 2010.^5^

While the direct health benefits of these programmes are clear, their broader health system implications and spill-over effects are less explored.^6^^–^^8^ For instance, controlling HIV infections can lead to reductions in risks associated with diarrhoeal disease,^9^ acute lower respiratory illness,^10^ childhood undernutrition and stunting,^11^ non-AIDS cancers,^12^ cardiovascular and cerebrovascular disease,^13^ depression,^14^ and maternal anaemia.^15^ Similarly, malaria control efforts can reduce the risk of conditions such as maternal anaemia, stillbirth and preterm birth.^16^ Additionally, tuberculosis control has been linked to a decrease in the incidence and severity of diabetes.^17^ Therefore, HIV/AIDS, tuberculosis and malaria programmes may have far-reaching health effects beyond their primary targets, potentially influencing health-care use and overall health system demand.

The evaluation of global health programmes traditionally relies on disease-specific models that focus solely on their individual health effects.^6^^,^^18^^–^^20^ This vertical approach, however, has limitations because it cannot capture the broader benefits of these interventions on other health conditions. Furthermore, the approach cannot account for individuals with multiple infections who might be counted in the deaths averted in multiple programmes. This situation can lead to an inflated sense of programme effectiveness, as someone potentially saved from one disease might still succumb to another within the same time.

To address these limitations, health system modelling is becoming important, as this approach can consider the real-world complexity of health-care systems and allow for the evaluation of programmes with a wider perspective.^21^ Modelling can quantify the programme’s effect on the entire spectrum of health conditions and health-care system use. Additionally, modelling can assess the overall needs of the health-care system associated with HIV/AIDS, tuberculosis and malaria programmes, both in the context of programme implementation and inaction.

As Malawi begins extensive health system reforms, a comprehensive health system modelling framework is clearly required that can provide a panoramic view of the health-care landscape.^22^^,^^23^

In this study, we use a whole health system model to quantify the effect that HIV/AIDS, tuberculosis and malaria programmes have had in Malawi between 2010 and 2019 by: (i) estimating the direct health benefits of the combined programmes; (ii) estimating the spill-over effects into other health conditions; (iii) quantifying the demands on the health system required to achieve these health benefits; and (iv) simulating the hypothetical demands on the health system had the three programmes not operated.

## Methods

### Thanzi la Onse model

We used the Thanzi la Onse model, which is a dynamic whole health system model that simulates the lifetime health of a representative Malawian population cohort.^24^ The model, detailed online with accessible source code, has been validated against reported data on disease burden, health-system engagement, availability of key consumables and service delivery volume.^25^^–^^28^ The model includes three main features: a realistic representation of the Malawian health system; a simulated population facing lifetime health hazards; and a statistical model of health system engagement. Disease modules track the onset, progression and care outcomes, while also capturing interactions between diseases based on underlying biological and clinical mechanisms. Individuals are assigned demographic characteristics based on survey data (e.g. age, sex and parity) and attributes related to lifestyle (e.g. education, wealth, obesity and smoking); health (e.g. noncommunicable and infectious diseases, mental health and reproductive or newborn health); and prior health-care use (based on treatment records and vaccination history).^27^ Detailed contraception choices, pregnancy, labour and delivery are modelled and calibrated to match available data.^29^

#### Health system capabilities

The capabilities of the health system depend on the distribution of health workforce, availability of essential medicines and diagnostic tools, and hospital bed capacity.^26^ The health system is divided into four levels: community (village clinics and outreach); primary (health-care centres); secondary (district hospitals); and tertiary (central hospitals). Each district has specific facilities for health-care delivery, with resources pooled within these levels. The provision of care is governed by the Malawi clinical guidelines, and health-care-seeking behaviour is determined by individual factors such as symptom severity, wealth, age and residence (rural or urban).^30^^–^^32^ The availability of key medicines and consumables is a critical factor in determining whether health-care appointments can proceed as planned; if these products are unavailable, patients may either return later or default on their care.

#### Disease modules

The framework covers neonatal and early childhood conditions, maternal conditions, communicable and noncommunicable diseases, and injuries, with additional risks modelled to align with estimates of the Global Burden of Disease Study.^33^ Our analysis focuses on three diseases^34^ (further information in the online repository).^35^

#### HIV

HIV risk in adults is modelled based on sexual contact, with varying risk factors including sex, wealth, education, voluntary medical male circumcision, pre-exposure prophylaxis use, or commercial sex work for females. Annual acquisition risk is calibrated using data from the Malawi population-based HIV impact assessment and UNAIDS (2010–2022).^2^^, ^^6^ Mother-to-child transmission is modelled across pregnancy, labour and breastfeeding, influenced by maternal ART and infant prophylaxis.

HIV testing can occur via provider-initiated testing during clinic visits, self-initiated testing, antenatal clinics, or routine testing for users of pre-exposure prophylaxis. Positive results prompt referrals for treatment, with viral suppression determined at the start of treatment. Individuals may stop or default from ART (due to stock-outs), seek to re-start later or undergo retesting as needed. Negative test results prompt a referral to other appropriate services such as behaviour change counselling, voluntary medical male circumcision or pre-exposure prophylaxis.

#### Tuberculosis

Active tuberculosis infections are based on untreated tuberculosis prevalence and individual risk factors such as Bacillus Calmette–Guérin vaccination, obesity, smoking, HIV status and use of isoniazid preventive therapy. Drug-sensitive and multidrug-resistant tuberculosis strains are modelled separately. Tuberculosis relapse risk is higher for people living with HIV. Mortality is associated with smear status and treatment success, and death occurs 1–5 months after disease onset.

Following Malawi clinical guidelines, diagnostic tools include sputum smear microscopy, GeneXpert, chest radiographs and clinical diagnosis, each with varying sensitivity and specificity. GeneXpert is prioritized for people living with HIV and relapse cases, chest radiographs are required for children younger than 5 years and clinical diagnosis is used if other tests are unavailable. Any positive results prompt immediate treatment, with confirmed cases of multidrug-resistant tuberculosis receiving specialized regimens. Isoniazid preventive therapy is given routinely to reduce active tuberculosis risk, with a 6-month course for tuberculosis contacts and a 36-month course for people living with HIV who are starting isoniazid preventive therapy with ART.

#### Malaria

An emulation model, based on an individual-based simulation calibrated to parasite prevalence data, captures malaria risk.^36^ This model includes age-specific, district-level incidences of asymptomatic, clinical and severe malaria in the presence of interventions such as indoor residual spraying and long-lasting insecticide-treated bednets. The model reflects the full dynamics of *Plasmodium falciparum* transmission, incorporating age and exposure-dependent immunity functions. By emulating this full transmission model, we account for complex factors including seasonality, decay of maternal immunity and exposure-dependent acquired immunity. Information on intervention coverage was taken from the Malaria Atlas Project.^37^

Malaria symptom onset occurs 7 days after infection, with clinical cases resolving through treatment or self-cure. Patients with severe disease require emergency care and may die within 7 days unless they receive appropriate treatment. The risks of clinical and severe malaria are increased in individuals with unsuppressed HIV infections, and mitigated by ART and co-trimoxazole use.

As per Malawi clinical guidelines, rapid diagnostic tests are used to confirm malaria diagnoses for all patients presenting with fever and are also offered through community outreach programmes to both symptomatic and asymptomatic individuals. Treatment is tailored to clinical severity, resolving symptoms and clearing parasitaemia within 7 days. Pregnant women attending antenatal care services are provided intermittent preventive treatment for malaria, which confers 6 weeks of protection against clinical disease.

#### Disease interactions

The Thanzi la Onse model includes a broad range of disease interactions (online repository).^35^ For untreated HIV, interactions include increased risks of tuberculosis (relative risk, RR 5.0 for active tuberculosis and RR 4.7 for relapse); altered tuberculosis presentation (35% smear-positive cases); and increased risks of clinical and severe malaria, particularly in pregnant women (RR 4.0 and RR 2.8, respectively). HIV also increases the risk of anaemia in pregnancy (RR 4.2), stunting in children (RR 1.5) and diarrhoeal diseases (RR 5.6 for moderate to severe diarrhoea and RR 5.0 for diarrhoeal mortality).

Tuberculosis-related interactions include the effects of diabetes, which increase the risk of active tuberculosis (RR 1.5), relapse (RR 1.9) and mortality (RR 1.5). Malaria significantly affects maternal health, raising risks of anaemia (RR 1.5), preterm labour (RR 3.1) and stillbirth (RR 1.8), with indirect effects such as preterm labour contributing to stunting and diarrhoeal disease.

Many conditions additionally share risk factors; for example, excessive alcohol use can exacerbate the risk or severity of cardiometabolic disorders, tuberculosis and mental health. Integrated health-care approaches are included in the Thanzi la Onse model, such as HIV testing offered through tuberculosis clinics, or bednets and intermittent preventive treatment of malaria given through antenatal care, reflecting the standard Malawi clinical guidelines.^30^ While our focus was on interactions involving HIV, tuberculosis and malaria, all disease interactions and care pathways are incorporated in the model.

### Model simulations

The model was started in January 2010 with a representative simulated population of 147 000 (one modelled person for every 100 so-called true individuals) and ran to December 2019. All code was executed in Python programming language 3.8 (Python Software Foundation, Wilmington, United States of America). We calibrated all disease modules to relevant data sources, including World Health Organization (WHO) reports (tuberculosis); UNAIDS data (HIV/AIDS); national surveillance data (HIV/AIDS and tuberculosis); and the Global Burden of Disease (HIV/AIDS, tuberculosis and malaria).^2^^,^^4^^,^^33^^,^^38^^–^^40^ We undertook no further calibration for this analysis. We performed five model runs for each scenario, with medians and 95% uncertainty intervals (UI) calculated for each output. We derived deaths and disability-adjusted life years (DALYs) averted from pairwise run comparisons, which we then summarized.

#### Definition of scenarios

We simulated the so-called actual scenario, depicting the services that were delivered throughout 2010–2019, along with four hypothetical counterfactual scenarios where HIV/AIDS, tuberculosis and malaria service packages were excluded individually or in combination for the duration of the simulation (Box 1). Under the hypothetical scenarios, when we excluded the HIV, tuberculosis and malaria services, only end-of-life or palliative care was provided for those conditions. We produced a summary of health status and health system use every year and calculated DALYs using published disability weights.^41^ We evaluated differences between scenarios through a pairwise comparison of corresponding runs (e.g. run 0 with run 0, run 1 with run 1, and so on). To ensure consistency, each run within a scenario started from the same initial conditions. We summarized the results using the median (UI). This method captures the variations between runs initialized with different random seeds and gives distinct results compared to a simple comparison of medians across draws. To calculate life expectancy at birth, we used standard life table methods with 5-year age groups up to age 90+ years and adjusted to separately account for age groups *<* 1 year and 1–4 years.^42^ We undertook a sensitivity analysis to quantify the marginal impact of HIV, tuberculosis and malaria programmes by contrasting the actual scenario with counterfactuals, where each programme in turn was systematically included (online repository).^35^

Box 1 **Services included in each scenario for the period 2010–2019 and percentage coverage in 2019, Malawi**

***Actual – all services available including for HIV/AIDS, tuberculosis and malaria, namely:***

- antiretroviral therapy: 85% in adults, 100% in children
- prevention of mother-to-child transmission: 95%
- pre-exposure prophylaxis for female sex workers: 5%
- infant prophylaxis: 80%
- voluntary medical male circumcision: 30%
- tuberculosis treatment: 75%
- tuberculosis preventive therapy: 67%
- BCG vaccination: 91%
- malaria treatment: 42%
- insecticide-treated bednets: 79%
- indoor residual spraying: 5%
- intermittent preventive therapy for pregnant women (2 doses): 76%
- co-trimoxazole: 89%^a^

***No HIV services – as for actual but with no HIV services, that is:***

- antiretroviral therapy, prevention of mother-to-child transmission, pre-exposure prophylaxis, infant prophylaxis and voluntary medical male circumcision: all 0%

***No tuberculosis services – as for actual but with no tuberculosis services, that is:***

- tuberculosis treatment, tuberculosis preventive therapy and BCG vaccination: all 0%
- contact investigation for index cases: none

***No malaria services – as for actual but with no malaria services, that is:***

- malaria treatment, insecticide-treated bednets, indoor residual spraying, intermittent preventive therapy for pregnant women and co-trimoxazole: all 0%^a^

**No HIV/AIDS, tuberculosis or malaria services, that is:**

- antiretroviral therapy, prevention of mother-to-child transmission, pre-exposure prophylaxis, infant prophylaxis, voluntary medical male circumcision, tuberculosis treatment, tuberculosis preventive therapy, BCG vaccination, malaria treatment, insecticide-treated bednets, indoor residual spraying, intermittent preventive therapy for pregnant women and co-trimoxazole: all 0%^a^
- contact investigation for index cases: none

BCG: Bacillus Calmette–Guérin; HIV/AIDS: human immunodeficiency virus/acquired immunodeficiency syndrome.^a^ Co-trimoxazole is given to people with HIV to prevent opportunistic infections; we only considered the effect on malaria incidence and severity.Note: All non-HIV/AIDS, tuberculosis and malaria services are consistently available as required across the scenarios, reflecting the true service availability throughout this period. Average coverage values in eligible populations are given for 2019 but they vary by district and year throughout the simulation.

#### Ethical approval

The Thanzi La Mawa project received ethical approval from the College of Medicine Malawi Research Ethics Committee (COMREC, P.09/23–0297) for the use of publicly accessible and anonymized secondary data. No data were used requiring individual informed consent. 

We followed the Guidelines for Accurate and Transparent Health Estimates Reporting (online repository).^35^

## Results

### Health impacts of the programmes

#### Direct health benefits

Excluding each set of services in turn and comparing population health with the actual scenario gave an estimate of the health gains attributable to each programme (online repository).^35^ The provision of HIV-related health-care services was estimated to have averted 31.95 million DALYs (95% UI: 31.60 to 32.74 million) due to HIV/AIDS, and 261 100 DALYs (95% UI: 135 900 to 417 100 million) due to tuberculosis during 2010–2019. Tuberculosis services directly prevented 5.48 million tuberculosis DALYs (95% UI: 5.32 to 6.06 million). Tuberculosis services also prevented a further 1.04 million HIV/AIDS DALYs (95% UI: 573 600 to 1.55 million) through increased HIV testing during tuberculosis care, and reduced tuberculosis incidence in people living with HIV, for whom tuberculosis onset marks the progression to AIDS. The provision of malaria services prevented 36.84 million DALYs (95% UI: 35.94 to 37.25 million) due to malaria.

**Fig. 1:**
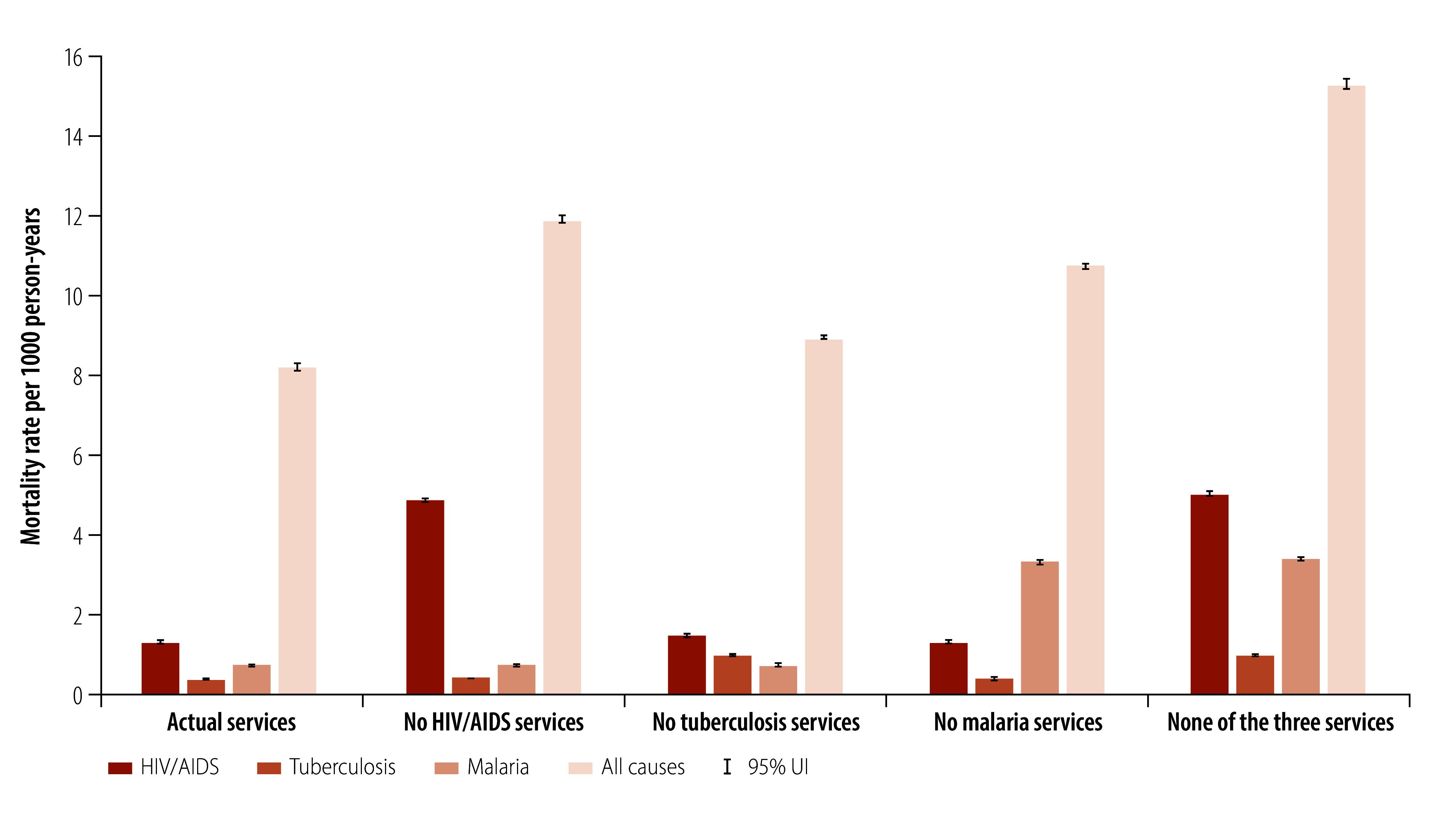
Mortality rates due to HIV/AIDS, tuberculosis, malaria and all causes by scenario, 2019

**Fig. 2:**
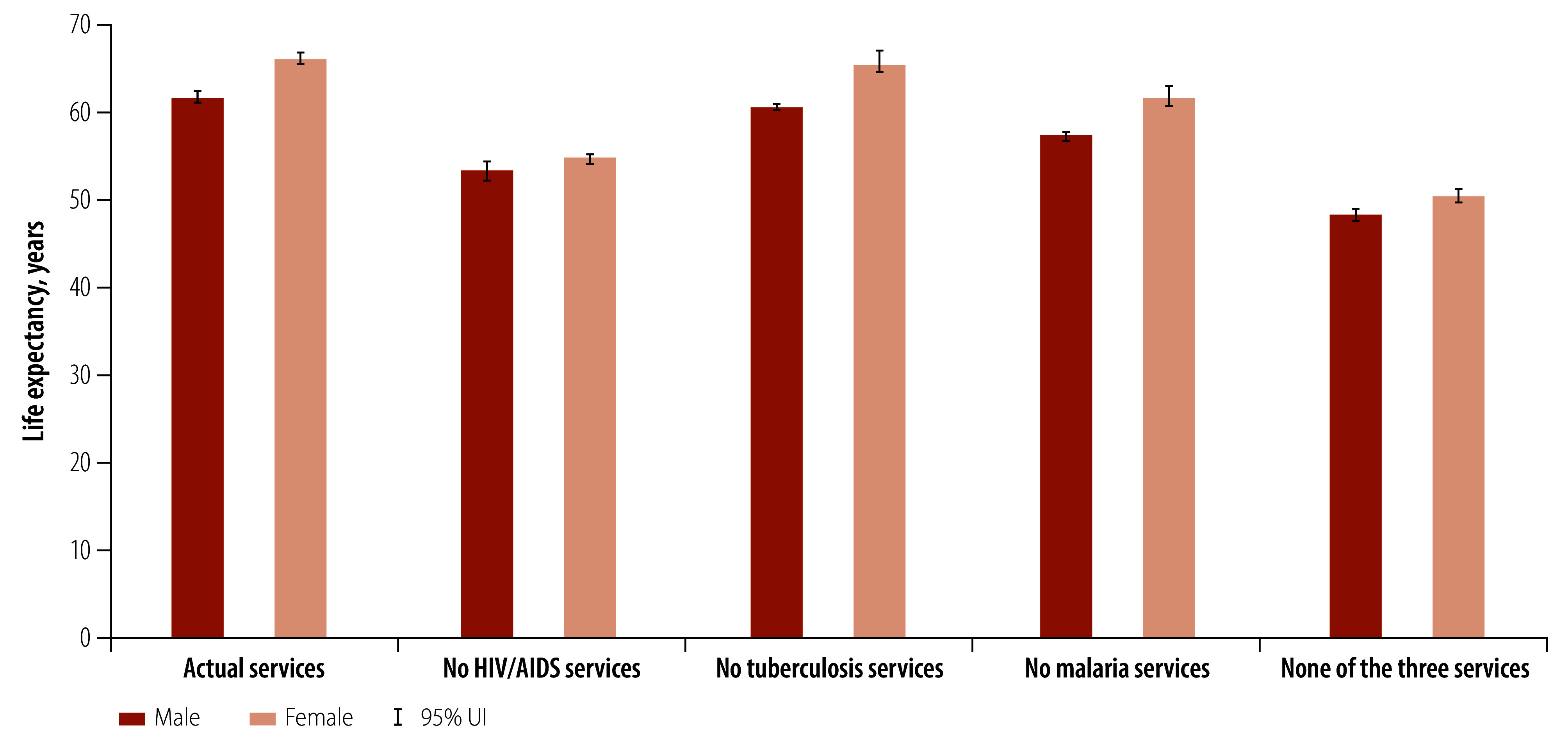
Estimated life expectancy at birth for males and females by scenario, 2019

Moving from single programme estimates to a joint programme estimate captures the cross-disease effects of the interventions (online repository).^35^ HIV/AIDS, tuberculosis and malaria programmes, when considered in combination, have prevented 579 300 (95% UI: 570 900 to 586 100), 94 200 (95% UI: 90 400 to 100 900) and 416 100 (95% UI: 414 000 to 420 800) deaths due to HIV/AIDS, tuberculosis and malaria, respectively (Fig. 1). In addition, jointly the programmes have averted 74.89 million HIV/AIDS, tuberculosis and malaria DALYs (95% UI: 74.18 to 75.16 million) over a 10-year period, increasing life expectancy at birth in 2019 for women by a median of 16.9 years (from a median of 50.4 with no HIV/AIDS, tuberculosis and malaria services to median 66.1 years in the actual scenario) and men by a median of 13.0 years (from 48.2 to 61.7 years; Fig. 2).

HIV/AIDS: human immunodeficiency virus/acquired immunodeficiency syndrome; UI: uncertainty interval.

Note: The error bars show the 95% UIs across the five model runs for each scenario.

HIV/AIDS: human immunodeficiency virus/acquired immunodeficiency syndrome; UI: uncertainty interval.

Note: The error bars show the 95% UIs across the five model runs for each scenario.

#### Spill-over effects

Our findings show the effects of HIV/AIDS, tuberculosis and malaria services on multiple health conditions. HIV services alone averted an estimated 1.99 million DALYs (95% UI: 1.37 to 2.31 million) associated with childhood diarrhoea, acute lower respiratory illness, non-AIDS cancers and tuberculosis between 2010 and 2019. Reductions in DALYs attributed to HIV/AIDS (1.04 million DALYs; 95% UI: 573 600 to 1.55 million) and acute lower respiratory illness (140 700 DALYs; 95% UI: −211 300 to 419 200) were associated with tuberculosis services. Additionally, malaria services decreased the risk of diabetes (42 200 DALYs; 95% UI: −22 200 to 107 400) and neonatal disorders (337 100 DALYs; 95% UI: −112 800 to 324 300). Although the UIs suggest these effects are not statistically significant, these reductions were observed in four out of five simulation runs.

Jointly, HIV/AIDS, tuberculosis and malaria services prevented 15 600 (95% UI: 4500 to 27 000) deaths not caused by these diseases. These services together reduced mortality rates for non-AIDS cancers (from 0.48 per person-year (95% UI: 0.46 to 0.49) to 0.44 per person-year (95% UI: 0.43 to 0.44)); childhood diarrhoea (from 0.25 per person-year (95% UI: 0.23 to 0.25) to 0.20 per person-year (95% UI: 0.18 to 0.21)); and acute lower respiratory illness (from 0.85 per person-year (95% UI: 0.82 to 0.89) to 0.80 per person-year (95% UI: 0.79 to 0.81)). Reductions in DALYs per person-year (median % change; 2.5th to 97.5th centile) were also noted for: kidney disease (−7.55; −18.50 to −1.34), acute lower respiratory illness (−6.79; −9.68 to −2.73) and measles (−8.67; −15.65 to −0.51; Table 1). The overall health benefits of HIV/AIDS, tuberculosis and malaria services are influenced by shifting demographics (online repository);^35^ reduced overall mortality rates and increased live birth rates increase person-years at risk for conditions such as chronic obstructive pulmonary disease, diabetes, stroke and kidney disease.

**Table 1:** Estimated impact of HIV/AIDS, tuberculosis and malaria services on DALYs per person-year and the associated health system appointments, 2010–2019, Malawi

Disease programme (disease)	Median % (2.5th to 97.5th centile) change in DALYs per person-year with HIV/AIDS, tuberculosis and malaria services^a^	No. of appointments, in thousands (2.5th to 97.5th centile)^b^	
		Actual scenario	No HIV/AIDS, tuberculosis and malaria services
Acute lower respiratory illness	−6.79 (−9.68 to −2.73)	6 630 (6 526 to 6 745)	6 817 (6 755 to 6 959)
Antenatal care (maternal disorders)	−5.83 (−31.00 to 9.95)	24 533 (24 291 to 24 769)	23 771 (23 619 to 24 008)
Bladder cancer	12.66 (−0.74 to 22.61)	203 (195 to 222)	179 (161 to 182)
Breast cancer	1.04 (−6.50 to 11.93)	204 (172 to 226)	207 (157 to 223)
Cardiometabolic disorders^c^		10 881 (10 677 to 11 125)	11 107 (10 965 to 11 181)
Diabetes	2.26 (−3.42 to 10.40)	NA	NA
Epilepsy	−9.11 (−13.73 to 4.74)	NA	NA
Heart disease	6.13 (−0.02 to 9.64)	NA	NA
Kidney disease	−7.55 (−18.50 to −1.34)	NA	NA
Stroke	2.26 (−7.38 to 11.43)	NA	NA
Contraception		345 124 (337 539 to 352 158)	331 995 (329 843 to 339 810)
Chronic obstructive pulmonary disorder	4.21 (−0.34 to 16.68)	317 (294 to 332)	300 (287 to 308)
Care during delivery	NA^d^	5 057 (5 006 to 5 121)	4 908 (4 874 to 4 938)
Neonatal disorders	−4.99 (−11.17 to 1.40)	NA^e^	NA^e^
Congenital birth defects	8.61 (−15.75 to 17.80)	NA^e^	NA^e^
Depression (self-harm)	3.73 (1.88 to 4.69)	325 (297 to 346)	324 (313 to 327)
Diarrhoea	−24.95 (−37.81 to −19.24)	17 076 (16 938 to 17 130)	34 356 (34 189 to 34 531)
Expanded Programme on Immunization	NA^d^	99 368 (98 596 to 100 378)	96 390 (95 772 to 97 011)
Epilepsy	−9.11 (−13.73 to 4.74)	25 157 (23 565 to 25 599)	25 615 (24 978 to 25 931)
HIV/AIDS	−234.27 (−242.02 to −224.29)	42 917 (42 805 to 43 186)	799 (793 to 808)
Malaria	−392.04 (−409.75 to −383.98)	98 178 (97 546 to 98 499)	541 (531 to 547)
Measles	−8.67 (−15.65 to −0.51)	2 618 (2 611 to 2 643)	2 530 (2 512 to 2 556)
Oesophageal cancer	−4.69 (−17.68 to 9.09)	174 (156 to 179)	166 (144 to 198)
Other adult cancers	−12.07 (−14.41 to −6.27)	436 (420 to 501)	448 (434 to 473)
Postnatal care	NA^d^	9 958 (9 876 to 10 136)	9 726 (9 700 to 9 841)
Prostate cancer	−19.40 (−24.45 to −1.63)	121 (110 to 130)	119 (104 to 125)
Road traffic injuries	2.57 (−6.58 to 4.33)	309 101 (294 622 to 319 903)	299 357 (295 570 to 308 391)
Schistosomiasis	0.80 (−2.99 to 2.34)	712 (700 to 715)	1 137 (1 114 to 1 160)
Tuberculosis	−174.11 (−183.23 to −149.13)	62 320 (62 278 to 62 526)	155 (153 to 161)
Undernutrition	NA^d^	326 (316 to 333)	630 (617 to 663)
First attendance emergency	NA^f^	8 699 (8 448 to 8 803)	9 087 (8 983 to 9 175)
First attendance non-emergency	NA^f^	33 523 (33 418 to 33 553)	53 998 (53 793 to 54 104)

DALY: disability-adjusted life year; HIV/AIDS: human immunodeficiency virus/acquired immunodeficiency syndrome; NA: not applicable.

^a^ Median percentage change in DALYs per person-year was calculated as: (No HIV/AIDS, tuberculosis and malaria scenario – actual scenario)/No HIV/AIDS, tuberculosis and malaria scenario x 100, representing the relative changes in disease burden due to operational HIV/AIDS, tuberculosis and malaria programmes. Negative values indicate HIV/AIDS, tuberculosis and malaria services have reduced the disease burden.

**^b^** The number of appointments (in thousands) classified by disease programme for the actual scenario and if no HIV/AIDS, tuberculosis and malaria services had been available. The data for the scenario of no HIV/AIDS, tuberculosis and malaria services relate to end-of-life or palliative care, which would not have any positive effect on health but would still be offered even in the absence of the HIV/AIDS, tuberculosis and malaria programmes. First attendance appointments refer to general outpatient appointments which are stratified into emergency and non-emergency appointments.

^c^ DALYs are classified into subcategories, while appointments are classified as cardiometabolic disorders.

^d^ No DALYs are incurred.

^e^ No specific appointment type.

^f^ These are appointment types and are not linked with a specific cause of DALYs.

Note: The median values of five runs are shown for each scenario along with the lower (2.5th centile) and upper (97.5th centile) bounds.

### Services required for programme delivery

**Fig. 3:**
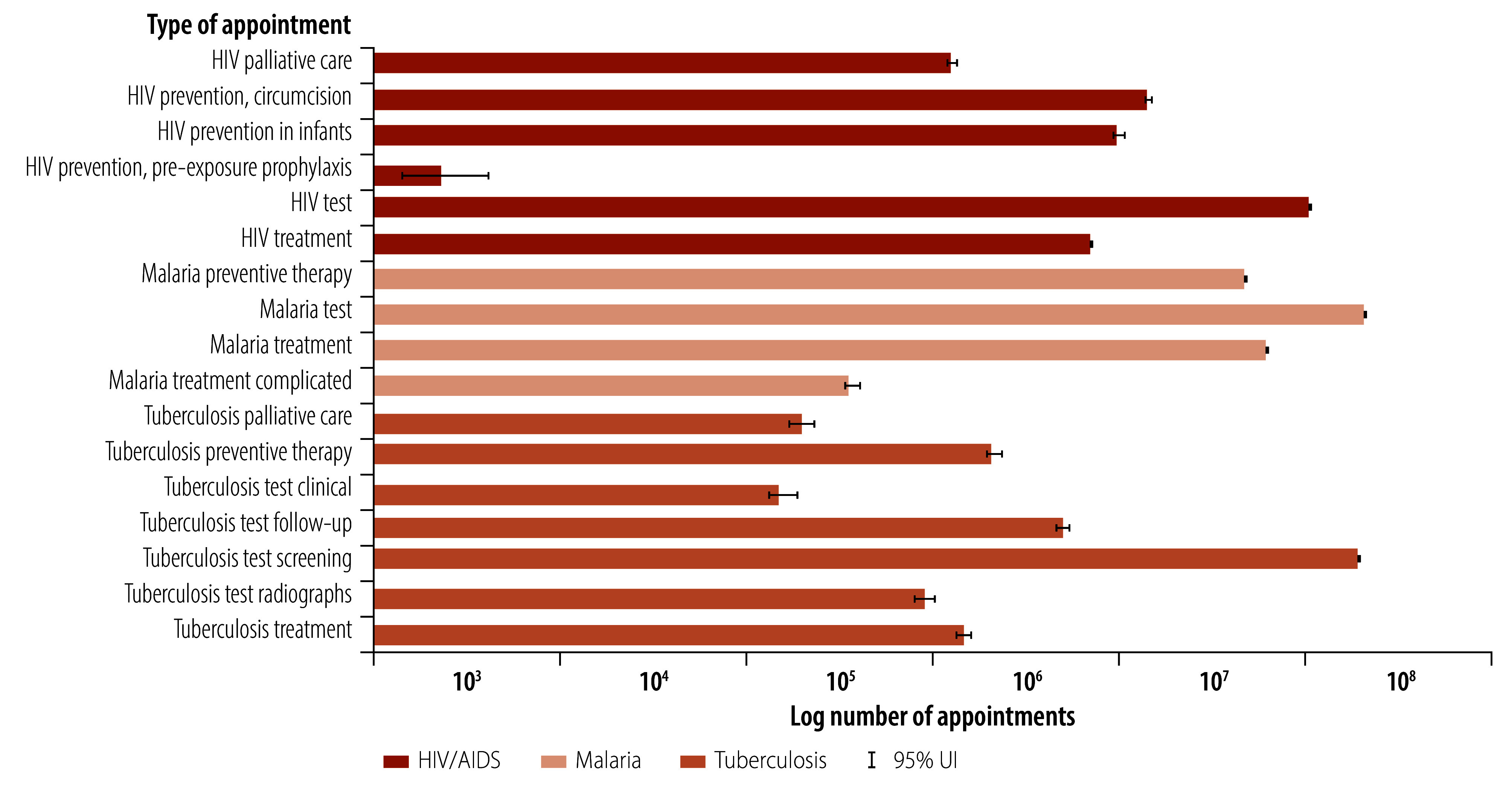
Estimated number of HIV/AIDS, tuberculosis and malaria appointments delivered by each programme between 2010 and 2019

HIV: human immunodeficiency virus; UI: uncertainty interval.

Notes: Some services are integrated within other appointments or delivered in the community. The error bars show the 95% UIs across the five model runs for each scenario.

During 2010–2019, 433.8 million (95% UI: 433.3 to 435.5 million) appointments were delivered for all health conditions, including community and outreach services, outpatient and inpatient care, and pharmacy and laboratory services, equivalent to 2.7 interactions per person per year (Table 2). Through these appointments, the following HIV/AIDS, tuberculosis and malaria services were delivered: 157.0 million (95% UI: 156.4 to 157.5 million) screening or diagnostic tests; 22.7 million (95% UI: 22.5 to 22.9 million) preventive services including voluntary medical male circumcision, isoniazid preventive therapy and pre-exposure prophylaxis; 23.2 million (95% UI: 23.1 to 23.2 million) treatment and follow-up appointments; and 558 700 (95% UI: 547 510 to 560 700) inpatient days (online repository).^35^ Fig. 3 shows the breakdown of appointments by disease area.

**Table 2:** Estimated numbers of appointments required to provide health-care services including and excluding HIV/AIDS, tuberculosis and malaria services

Type of appointment	Appointment numbers (2.5th to 97.5th centile) required in the actual scenario, in millions	Appointment numbers (2.5th to 97.5th centile) required in the no HIV/AIDS, tuberculosis and malaria scenario, in millions
Outpatient	344.21 (343.79 to 345.88)	223.76 (223.12 to 224.20)
Laboratory^a^	2.95 (2.92 to 3.02)	0.96 (0.94 to 0.97)
Pharmacy	6.13 (6.02 to 6.32)	5.85 (5.73 to 5.94)
Inpatient	80.61 (80.25 to 80.91)	89.77 (89.67 to 90.53)

HIV/AIDS: human immunodeficiency virus/acquired immunodeficiency syndrome.

^a^ Laboratory services are currently only included in the model with HIV/AIDS, tuberculosis and malaria services.

During this time, HIV/AIDS, tuberculosis and malaria programmes accounted for 18.5% (95% UI: 18.2 to 18.6%) of all health-care service interactions, decreasing from 22.3% (95% UI: 22.0 to 22.7%) in 2010 to 16.0% (95% UI: 15.3 to 16.2%) in 2019 (online repository).^35^ Without these programmes, the perceived reduction in health-system usage would be offset by the needs of untreated patients. Specifically, there would have been an estimated 120.7 million fewer outpatient appointments, 2.0 million fewer laboratory services and 323 500 fewer pharmacy visits, but 9.4 million additional hospital admissions and inpatient days, primarily for severe malaria and advanced HIV disease (Table 2).

Without HIV/AIDS, tuberculosis and malaria programmes, the health worker time required would have been 201.49 million patient-facing hours, only slightly lower than the 243.04 million hours used. Therefore, the adjusted cost in health worker time required by HIV/AIDS, tuberculosis and malaria programmes over 10 years was 41.5 million hours, or 17.1% (95% UI: 15.9 to 17.4%) of all patient-facing time (Table 3). Most of this difference was due to reduced demands on health surveillance assistants, who perform malaria tests and other community services.

**Table 3:** Estimated hours of each type of health worker required to provide health-care services including and excluding HIV/AIDS, tuberculosis and malaria services

Type of health worker	Hours (2.5th to 97.5th centile) required in the actual scenario, in millions	Hours (2.5th to 97.5th centile) required in the no HIV/AIDS, tuberculosis and malaria scenario, in millions	% (95% UI) reduction in health-worker time in the no HIV/AIDS, tuberculosis and malaria scenario^a^
Clinicians	94.22 (93.24 to 94.53)	81.94 (81.78 to 82.94)	12.93 (11.08 to 13.40)
Health surveillance assistants	6.40 (6.38 to 6.45)	0.05 (0.05 to 0.05)	99.27 (99.4 to 99.28)
Laboratory technicians	0.54 (0.53 to 0.55)	0 (0 to 0)^b^	100.00 (100.00 to 100.00)
Mental health workers	0.07 (0.06 to 0.07)	0.06 (0.06 to 0.07)	2.22 (–7.87 to 10.42)
Nurses and midwives	113.74 (113.61 to 114.14)	96.20 (96.11 to 97.08)	15.40 (14.59 to 15.67)
Pharmacists	27.58 (27.42 to 27.69)	22.82 (22.76 to 22.85)	17.39 (16.71 to 17.74)
Radiographers	0.49 (0.48 to 0.49)	0.41 (0.41 to 0.42)	15.19 (12.93 to 15.69)
**Total**	**243.04 (241.72 to 243.43)**	**201.49 (201.16 to 203.35)**	**17.07 (15.92 to 17.40)**

HIV/AIDS: human immunodeficiency virus/acquired immunodeficiency syndrome; UI: uncertainty interval.

^a^ We calculated the percentage reduction in hours through a pairwise comparison run-by-run when comparing the hypothetical scenario excluding HIV/AIDS, tuberculosis and malaria services to the actual scenario.

^b^ Laboratory services are currently only included in the model with HIV/AIDS, tuberculosis and malaria services.

## Discussion

HIV/AIDS, tuberculosis and malaria programmes accounted for almost a fifth of all health-system interactions in Malawi between 2010 and 2019, delivering significant health gains while requiring substantial clinical, nurse and pharmacist time. These programmes considerably reduced the burdens of the targeted diseases while decreasing susceptibility to co-morbidities, such as acute lower respiratory illness, diarrhoeal diseases and non-AIDS cancers. DALYs averted are not mutually exclusive. For example, both the HIV/AIDS and tuberculosis programmes can reduce HIV/AIDS- and tuberculosis-related DALYs, with similar interactions considered for co-infections such as malaria and HIV, which ensures that the effects of co-infection are captured without double-counting.

Empirical studies show significant gains in life expectancy due to ART (10-year increase in adults),^43^ malaria elimination (6-year increase in children)^44^ and overall health improvements (10-year increase).^45^ We show that HIV/AIDS, tuberculosis and malaria programmes implemented from pregnancy and delivery through childhood and into adulthood have prevented more than 75 million DALYs within 10 years, increasing life expectancy at birth by 13.0 years in men and 16.9 years in women, and reducing inpatient admissions by nearly 1 million (9.4 million bed-days). This increased life expectancy also exposes people to the risk of conditions such as depression and heart disease, highlighting the need for integrated services that address both communicable and noncommunicable diseases.

Despite robust data for HIV/AIDS, tuberculosis and malaria programme outcomes, we found gaps in key indicators and underreporting which created uncertainty. Expert input helped ensure consistency between our model, existing models and programme data. The fixed 2018 estimate for the availability of consumables may not fully reflect changes from 2010 or account for evolving treatment guidelines.^46^ Additionally, a fixed human resource footprint could overestimate the service delivery time required. Although the model prioritizes clarity and focuses on health outcomes and system delivery, it underrepresents broader impacts such as workforce training, infrastructure and supply chain management. As such the model likely underestimates the overall benefits of HIV/AIDS, tuberculosis and malaria programmes.^47^^,^^48^ While key disease interactions are captured, second-order effects (e.g. malaria and acute lower respiratory illness) are excluded. HIV/AIDS, tuberculosis and malaria interventions, such as ART, enhanced tuberculosis control and malaria prevention, provide cumulative benefits; for instance, malaria interventions reduce mortality in children younger than 5 years within 2.5 years,^49^ and so the full benefit of the programmes may not be captured here.

Ongoing strategies within HIV/AIDS, tuberculosis and malaria programmes, including active tuberculosis case finding, community testing and treatment adherence clubs, strengthen programme outcomes; they achieved 90% tuberculosis treatment success rates in 2023 and reduced AIDS deaths to about 11 000 annually.^2^^,^^8^ High-risk populations, including health workers, face high tuberculosis rates due to ineffective infection control and overcrowded facilities, adding pressure to an already strained system.^50^ Protecting these populations would further reduce health system pressure.

Service delivery improvements have reduced the burden on health systems while increasing the impact on health, with only 17.1% more of health worker time contributing to more than a 10-year increase in life expectancy. This outcome highlights the efficiency of HIV/AIDS, tuberculosis and malaria programmes. Further gains in efficiency could reduce overall demands on the health system and hence support sustainability. When global health funding decisions are being made, a holistic understanding of programme effectiveness and resource implications should guide prioritization to maximize overall health impact.

## References

[1] Results report. Geneva: The Global Fund to Fight AIDS Tuberculosis and Malaria; 2023. Available from: https://archive.theglobalfund.org/media/13263/archive_2023-results-report_report_en.pdf [cited 2023 Nov 2].

[2] AIDSinfo [internet]. Geneva: Joint United Nations Programme on HIV/AIDS (UNAIDS); 2023. Available from: https://aidsinfo.unaids.org/ [cited 2023 Nov 2].

[3] van Lettow M, Landes M, van Oosterhout JJ, Schouten E, Phiri H, Nkhoma E, et al. Prevention of mother-to-child transmission of HIV: a cross-sectional study in Malawi. Bull World Health Organ. 2018 Apr 1;96(4):256–65. 10.2471/BLT.17.203265 29695882 PMC5872011

[4] Ministry of Health Malawi. National tuberculosis control programme annual report 2022. Lilongwe: Ministry of Health Malawi; 2022.

[5] Global Health Observatory (GHO). Malaria burden data [internet]. Geneva: World Health Organization; 2023. Available from: https://www.who.int/data/gho/data/themes/topics/topic-details/GHO/malaria-cases-deaths [cited 2023 Nov 2].

[6] Payne D, Wadonda-Kabondo N, Wang A, Smith-Sreen J, Kabaghe A, Bello G, et al.; MPHIA Study Team. Trends in HIV prevalence, incidence, and progress towards the UNAIDS 95-95-95 targets in Malawi among individuals aged 15-64 years: population-based HIV impact assessments, 2015-16 and 2020-21. Lancet HIV. 2023 Sep;10(9):e597–605. 10.1016/S2352-3018(23)00144-3 37586390 PMC10542580

[7] Malawi multiple indicator cluster survey 2019–2020. Survey findings report. Zomba: National Statistical Office of Malawi; 2021.

[8] Global tuberculosis report 2022. Geneva: World Health Organization; 2022. Available from: https://iris.who.int/handle/10665/363752 [cited 2023 Nov 2].

[9] Acácio S, Nhampossa T, Quintó L, Vubil D, Sacoor C, Kotloff K, et al. The role of HIV infection in the etiology and epidemiology of diarrheal disease among children aged 0-59 months in Manhiça District, Rural Mozambique. Int J Infect Dis. 2018 Aug;73:10–7. 10.1016/j.ijid.2018.05.012 29852260 PMC6069671

[10] Jackson S, Mathews KH, Pulanic D, Falconer R, Rudan I, Campbell H, et al. Risk factors for severe acute lower respiratory infections in children: a systematic review and meta-analysis. Croat Med J. 2013 Apr;54(2):110–21. 10.3325/cmj.2013.54.110 23630139 PMC3641871

[11] Abate BB, Aragie TG, Tesfaw G. Magnitude of underweight, wasting and stunting among HIV positive children in East Africa: A systematic review and meta-analysis. PLoS One. 2020 Sep 17;15(9):e0238403. 10.1371/journal.pone.0238403 32941443 PMC7498078

[12] Shiels MS, Cole SR, Kirk GD, Poole C. A meta-analysis of the incidence of non-AIDS cancers in HIV-infected individuals. J Acquir Immune Defic Syndr. 2009 Dec;52(5):611–22. 10.1097/QAI.0b013e3181b327ca 19770804 PMC2790038

[13] Islam FM, Wu J, Jansson J, Wilson DP. Relative risk of cardiovascular disease among people living with HIV: a systematic review and meta-analysis. HIV Med. 2012 Sep;13(8):453–68. 10.1111/j.1468-1293.2012.00996.x 22413967

[14] Ciesla JA, Roberts JE. Meta-analysis of the relationship between HIV infection and risk for depressive disorders. Am J Psychiatry. 2001 May;158(5):725–30. 10.1176/appi.ajp.158.5.725 11329393

[15] Adamu AL, Crampin A, Kayuni N, Amberbir A, Koole O, Phiri A, et al. Prevalence and risk factors for anemia severity and type in Malawian men and women: urban and rural differences. Popul Health Metr. 2017 Mar 29;15(1):12. 10.1186/s12963-017-0128-2 28356159 PMC5371260

[16] Kalilani L, Mofolo I, Chaponda M, Rogerson SJ, Meshnick SR. The effect of timing and frequency of Plasmodium falciparum infection during pregnancy on the risk of low birth weight and maternal anemia. Trans R Soc Trop Med Hyg. 2010 Jun;104(6):416–22. 10.1016/j.trstmh.2010.01.013 20207387 PMC4844554

[17] Huangfu P, Ugarte-Gil C, Golub J, Pearson F, Critchley J. The effects of diabetes on tuberculosis treatment outcomes: an updated systematic review and meta-analysis. Int J Tuberc Lung Dis. 2019 Jul 1;23(7):783–96. 10.5588/ijtld.18.0433 31439109

[18] Kerr CC, Stuart RM, Gray RT, Shattock AJ, Fraser-Hurt N, Benedikt C, et al. Optima: a model for HIV epidemic analysis, program prioritization, and resource optimization. J Acquir Immune Defic Syndr. 2015 Jul 1;69(3):365–76. 10.1097/QAI.0000000000000605 25803164

[19] Griffin JT, Bhatt S, Sinka ME, Gething PW, Lynch M, Patouillard E, et al. Potential for reduction of burden and local elimination of malaria by reducing Plasmodium falciparum malaria transmission: a mathematical modelling study. Lancet Infect Dis. 2016 Apr;16(4):465–72. 10.1016/S1473-3099(15)00423-5 26809816 PMC5206792

[20] Houben RMGJ, Menzies NA, Sumner T, Huynh GH, Arinaminpathy N, Goldhaber-Fiebert JD, et al. Feasibility of achieving the 2025 WHO global tuberculosis targets in South Africa, China, and India: a combined analysis of 11 mathematical models. Lancet Glob Health. 2016 Nov;4(11):e806–15. 10.1016/S2214-109X(16)30199-1 27720688 PMC6375908

[21] Cassidy R, Singh NS, Schiratti PR, Semwanga A, Binyaruka P, Sachingongu N, et al. Mathematical modelling for health systems research: a systematic review of system dynamics and agent-based models. BMC Health Serv Res. 2019 Nov 19;19(1):845. 10.1186/s12913-019-4627-7 31739783 PMC6862817

[22] Ministry of Health Malawi. Government of the Republic of Malawi health sector strategic plan III 2023–2030. Reforming for universal health coverage. Lilongwe: Ministry of Health Malawi; 2023.

[23] Verguet S, Feldhaus I, Jiang Kwete X, Aqil A, Atun R, Bishai D, et al. Health system modelling research: towards a whole-health-system perspective for identifying good value for money investments in health system strengthening. BMJ Glob Health. 2019 Apr 28;4(2):e001311. 10.1136/bmjgh-2018-001311 31139448 PMC6509611

[24] Hallett TB, Mangal TD, Tamuri AU, Arinaminpathy N, Cambiano V, Chalkley M, et al. Estimates of resource use in the public-sector health-care system and the effect of strengthening health-care services in Malawi during 2015-19: a modelling study (Thanzi La Onse). Lancet Glob Health. 2025 Jan;13(1):e28–37. 10.1016/S2214-109X(24)00413-3 39549712

[25] The Thanzi La Onse Model, v0.1. Meyrin: Zenodo; 2023. 10.5281/zenodo.10144015

[26] Mohan S, Mangal TD, Colbourn T, Chalkley M, Chimwaza C, Collins JH, et al. Factors associated with medical consumable availability in level 1 facilities in Malawi: a secondary analysis of a facility census. Lancet Glob Health. 2024 Jun;12(6):e1027–37. 10.1016/S2214-109X(24)00095-0 38762283

[27] Malawi demographic and health survey 2015–16. Zomba: National Statistical Office & Rockville, MD: ICF; 2017. Available from: https://dhsprogram.com/pubs/pdf/FR319/FR319.pdf [cited 2025 Feb 12].

[28] She B, Mangal TD, Prust ML, Heung S, Chalkley M, Colbourn T, et al. Health workforce needs in Malawi: analysis of the Thanzi La Onse integrated epidemiological model of care. Hum Resour Health. 2024 Sep 27;22(1):66. 10.1186/s12960-024-00949-2 39334127 PMC11437829

[29] Colbourn T, Janoušková E, Li Lin I, Collins J, Connolly E, Graham M, et al. Modeling contraception and pregnancy in Malawi: a Thanzi La Onse mathematical modeling study. Stud Fam Plann. 2023 Dec;54(4):585–607. 10.1111/sifp.12255 38129327 PMC10941698

[30] Ministry of Health Malawi. Malawi standard treatment guidelines (MSTG). 5th ed. Lilongwe: Ministry of Health Malawi; 2015.

[31] Ng’ambi W, Mangal T, Phillips A, Colbourn T, Mfutso-Bengo J, Revill P, et al. Factors associated with healthcare seeking behaviour for children in Malawi: 2016. Trop Med Int Health. 2020 Dec;25(12):1486–95. 10.1111/tmi.13499 32981174

[32] Ng’ambi W, Mangal T, Phillips A, Colbourn T, Nkhoma D, Mfutso-Bengo J, et al. A cross-sectional study on factors associated with health seeking behaviour of Malawians aged 15+ years in 2016. Malawi Med J. 2020 Dec;32(4):205–12. 34457205 10.4314/mmj.v32i4.5 PMC8364791

[33] GBD 2017 Causes of Death Collaborators; GBD 2017 Causes of Death Collaborators. Global, regional, and national age-sex-specific mortality for 282 causes of death in 195 countries and territories, 1980-2017: a systematic analysis for the Global Burden of Disease Study 2017. Lancet. 2018 Nov 10;392(10159):1736–88. 10.1016/S0140-6736(18)32203-7 30496103 PMC6227606

[34] Mangal TD, Mohan S, Colbourn T, Collins JH, Graham M, Jahn A, et al. Assessing the effect of health system resources on HIV and tuberculosis programmes in Malawi: a modelling study. Lancet Glob Health. 2024 Oct;12(10):e1638–48. 10.1016/S2214-109X(24)00259-6 39304236

[35] Mangal TD, Molaro M, Nkhoma D, Colbourn T, Collins JH, Janoušková E, et al. Modelling health outcomes of a decade of HIV, malaria and tuberculosis initiatives, Malawi. Amsterdam: Mendeley; 2025. 10.17632/pwnbpvd44p.1 PMC12067010 40357264

[36] Griffin JT, Hollingsworth TD, Okell LC, Churcher TS, White M, Hinsley W, et al. Reducing Plasmodium falciparum malaria transmission in Africa: a model-based evaluation of intervention strategies. PLoS Med. 2010 Aug 10;7(8):e1000324. 10.1371/journal.pmed.1000324 20711482 PMC2919425

[37] Malaria atlas project data platform [internet]. Perth: Malaria Atlas Project; 2023. Available from: https://data.malariaatlas.org/trends?year=2022&metricGroup=Malaria&geographicLevel=admin0&metricSubcategory=Pf&metricType=rate&metricName=incidence [cited 2025 Feb 12].

[38] Global tuberculosis database. Tuberculosis profile: Malawi. Geneva: World Health Organization; 2023. Available from: https://www.who.int/teams/global-tuberculosis-programme/data [cited 2025 Feb 12].

[39] Malawi population–based HIV impact assessment (MPHIA) 2015–16. Lilongwe: Ministry of Health Malawi; 2018.

[40] Malawi population-based HIV impact assessment (MPHIA 2020–2021): final report. Lilongwe: Ministry of Health Malawi; 2022.

[41] Salomon JA, Haagsma JA, Davis A, de Noordhout CM, Polinder S, Havelaar AH, et al. Disability weights for the Global Burden of Disease 2013 study. Lancet Glob Health. 2015 Nov;3(11):e712–23. 10.1016/S2214-109X(15)00069-8 26475018

[42] Chiang CL. The life table and its construction. In: Introduction to stochastic processes in biostatistics. New York: John Wiley & Sons; 1968:198–214.

[43] Price AJ, Glynn J, Chihana M, Kayuni N, Floyd S, Slaymaker E, et al. Sustained 10-year gain in adult life expectancy following antiretroviral therapy roll-out in rural Malawi: July 2005 to June 2014. Int J Epidemiol. 2017 Apr 1;46(2):479–91. 28338707 10.1093/ije/dyw208 PMC5813794

[44] Bawah AA, Binka FN. How many years of life could be saved if malaria were eliminated from a hyperendemic area of northern Ghana? Am J Trop Med Hyg. 2007 Dec;77(6) Suppl:145–52. 10.4269/ajtmh.2007.77.145 18165487

[45] Tracking universal health coverage in the WHO African Region. Geneva: World Health Organization; 2022. Available from: https://iris.who.int/handle/10665/361229 [cited 2023 Nov 2].

[46] Berman L, Prust ML, Maungena Mononga A, Boko P, Magombo M, Teshome M, et al. Using modeling and scenario analysis to support evidence-based health workforce strategic planning in Malawi. Hum Resour Health. 2022 Apr 18;20(1):34. 10.1186/s12960-022-00730-3 35436946 PMC9014573

[47] Rasschaert F, Pirard M, Philips MP, Atun R, Wouters E, Assefa Y, et al. Positive spill-over effects of ART scale up on wider health systems development: evidence from Ethiopia and Malawi. J Int AIDS Soc. 2011 Jul 6;14(Suppl 1) Suppl 1:S3. 10.1186/1758-2652-14-S1-S3 21967809 PMC3194148

[48] Bekker L-G, Alleyne G, Baral S, Cepeda J, Daskalakis D, Dowdy D, et al. Advancing global health and strengthening the HIV response in the era of the Sustainable Development Goals: the International AIDS Society-Lancet Commission. Lancet. 2018 Jul 28;392(10144):312–58. 10.1016/S0140-6736(18)31070-5 30032975 PMC6323648

[49] Yé Y, Eisele TP, Eckert E, Korenromp E, Shah JA, Hershey CL, et al. Framework for evaluating the health impact of the scale-up of malaria control interventions on all-cause child mortality in sub-Saharan Africa. Am J Trop Med Hyg. 2017 Sep;97(3_Suppl) Suppl:9–19. 10.4269/ajtmh.15-0363 28990923 PMC5619929

[50] Alele FO, Franklin RC, Emeto TI, Leggat P. Occupational tuberculosis in healthcare workers in sub-Saharan Africa: A systematic review. Arch Environ Occup Health. 2019;74(3):95–108. 10.1080/19338244.2018.1461600 29702035

